# Direct interactions between ENaC gamma subunit and ClCN2 in cystic fibrosis epithelial cells

**DOI:** 10.14814/phy2.12264

**Published:** 2015-01-27

**Authors:** Katherine R. Henry, Seakwoo Lee, Douglas Walker, Pamela L. Zeitlin

**Affiliations:** Department of Pediatrics, The Johns Hopkins University School of Medicine, Baltimore, Maryland, USA

**Keywords:** Chloride channel N2, chloride secretion, cystic fibrosis, epithelial sodium channel, sodium absorption, ubiquitination

## Abstract

Cystic fibrosis (CF) is a lethal disease caused by mutations in the chloride channel CFTR gene. The disease is characterized by decreased chloride secretion and unregulated sodium absorption through the epithelial sodium channel (ENaC) in the airway epithelium and other affected organs. We hypothesize that a non‐CFTR alternative chloride channel ClCN2 can be activated to negatively regulate ENaC in CF epithelial cell cultures. We identified a novel interaction between ClCN2 and the ENaC*γ* subunit in CF airway epithelial cells and show that the upregulation of ClCN2 leads to decreased expression of ENaC*γ* via a K63 ubiquitination mechanism. These regulatory effects of ClCN2 on ENaC*γ* appear to be dependent on the CBS‐1 domain located within the c‐terminus of ClCN2, which is necessary for the targeting of ClCN2 to the apical surface. In sum, these results suggest the ability of ClCN2 to negatively regulate sodium absorption through ENaC, supporting its role as a therapeutic target for the treatment of CF.

## Abstract (precis)

Cystic Fibrosis is caused by mutations in the chloride channel CFTR gene which secondarily increases sodium reabsorption in the airways. ClCN2 is an epithelial chloride channel in the airways that can be activated in CF. We show an interaction between ClCN2 and the epithelial sodium channel subunit gamma that affects subunit expression and targeting to the plasma membrane.

## Introduction

Cystic fibrosis (CF) is an autosomal recessive disease caused by mutations in the cystic fibrosis transmembrane regulator (CFTR) gene (Riordan 1999). Normally, in healthy individuals active CFTR promotes an optimal balance between sodium (Na^+^) absorption and chloride (Cl^−^) secretion in the epithelial cells of secretory organs (Riordan 1999; Berdiev et al. 2007; Gentzsch et al. 2010). In airways this balance of sodium and chloride promotes normal periciliary hydration and mucociliary defense.

In normal epithelium, sodium absorption occurs via the epithelial sodium channel (ENaC) and is down regulated by CFTR (Riordan 1999; Berdiev et al. 2007; Gentzsch et al. 2010). In the lungs, ENaC is composed of three subunits, alpha (*α*), beta (*β*), and gamma (*γ*) (Zhou et al. 2011). Although the three subunits act together in unison, the regulation of each subunit may vary (Eaton et al. 2010). In the absence of CFTR activity, Na^+^ absorption is augmented, leading to dehydration of the mucous membrane. Furthermore, CFTR may help to maintain airway regulation of paracellular permeability of tight junctions (Lesimple et al. 2010). Indeed, in patients with CF, there is a marked disruption of tight junction formation in intestinal cells, creating a leaky barrier between the cells resulting in unwanted flow of ions and molecules between the cells. Consequently, this unchecked permeability may then contribute to the development of bacterial infections and disease severity (Zulianello et al. 2006).

In order to restore the functions lost by mutant CFTR, some therapeutic treatments are focusing on identifying alternative chloride channels (Flume and Van Devanter 2012). Ideally, to be most effective, a target channel would have similar expression patterns to CFTR, as well as an ability to regulate Na^+^ absorption by ENaC and restore cell barriers by promoting the formation of tight junctions. One such chloride channel, ClCN2 is a volume and pH activated channel with a similar expression pattern to CFTR in both intestinal and lung epithelia and has been shown to effectively rescue Cl^−^ secretion in the absence of CFTR (Murray et al. 1995, 1996; Schwiebert et al. 1998; Blaisdell et al. 1999). Activation of ClCN2 has already been achieved by the prostone drug lubiprostone (Amitiza) (Lacy and Levy 2007; MacDonald et al. 2008). Indeed, we have previously shown that Lubiprostone is able to rescue Cl^−^ secretion in CFTR knockout mice, supporting its role in activating ClCN2 (Schiffhauer et al. 2013) through a CFTR‐independent mechanism. Lubiprostone is a gastrointestinal targeting bicyclic fatty acid, which is currently used to treat constipation by restoring chloride secretion in the gut (Cuppoletti et al. 2004; McKeage et al. 2006). Furthermore, ClCN2 agonists used in the intestinal epithelium are shown to increase barrier formation and function in animal models (Moeser et al. 2007, 2008; Cuppoletti et al. 2012; Nighot et al. 2012).

The ability of ClCN2 to restore Cl^−^ secretion and barrier function is increasingly documented; however, its ability to regulate ENaC is unknown. Although an exact mechanism for the regulation of ENaC by CFTR has yet to be defined, studies suggest the potential for both an indirect and direct mechanism of action (Briel et al. 1998; Berdiev et al. 2007; Gentzsch et al. 2010; Rubenstein et al. 2011). Therefore, we sought to determine the effectiveness of increased ClCN2 targeting to the apical surface by exploiting potential targeting sequences in the ClCN2 c‐terminus (Pena‐Munzenmayer et al. 2005), in restoring CFTR‐ like functions in human lung epithelial cell lines, specifically, the physical regulation of ENaC at the apical surface in lung epithelium by ClCN2. If successful, the targeted increase in ClCN2 or ClCN2‐mediated mechanisms may provide additional therapeutic targets for the successful treatment of CF.

## Materials and Methods

### Cell culture

All studies were conducted in human cystic fibrosis bronchial epithelial cells (CFBE). Cells were grown to confluence in MEM media (Gibco, Grand Island, NY) containing 10% FBS, 1% antibiotic at 37°C, 5% CO_2_ and allowed to polarize at an air surface liquid interface on polyester Transwell inserts (Corning, Tewskbury, MA) as determined by transepithelial resistance using an Ohm resistance reader. Inserts were studied at a minimum resistance of 280 Ω.

### Site‐directed mutagenesis

Site‐directed mutagenesis was performed using PCR with forward and reverse primers as previously described (Lee et al. 2014) to create truncated ClCN2 mutants. Mutants were inserted into a previously constructed FLAG‐tagged ClCN2 expression vector and verified using sequence confirmation.

### Transfection

Cells were transfected with 5 *μ*g wild‐type or mutant ClCN2 cDNA constructs in Opti‐MEM media (Gibco) per well, as per the Lipofectamine^®^2000 protocol (Life Technologies, Grand Island, NY). Cells were harvested 18 h posttransfection for further analysis. Transfection efficiency was determined by calculating the ratio of cells that show IF staining for FLAG to total nuclei stained per mutant in single frame scans from a minimum of four separate transfections and showed no statistical variation between the four FLAG‐tagged mutants.

### Biotinylation assay

Cell surface analysis of ENaC*γ* and FLAG‐tagged ClCN2 was performed as previously described (Singh et al. 2008). Following transfection, cells on the apical surface were labeled with a 10 mmol/L Sulfo‐NHS‐SS Biotin solution (Thermo Scientific, Waltham, MA) for 30 min on ice. Biotinylated proteins were immunoprecipitated using Avidin Agarose beads (Thermo Scientific) and analyzed by a standard immunoblot protocol.

### Immunoblot analysis

Immunoblot analysis was performed by standard protocol. Briefly, cells were lysed using RIPA buffer and protein concentration was determined by Pierce BCA protein assay kit (Thermo Fisher, Waltham MA). Electrophoresis and Western transfer were performed via standard protocol, and 0.2 *μ*m nitrocellulose membrane (Bio‐Rad, Hercules, CA) was blocked using 5% Blotting Grade Blocker (Bio‐Rad). Primary antibodies for FLAG (mouse monoclonal, Sigma, St. Louis, MO) ENaC*γ* (rabbit polyclonal, Abcam, Cambridge, MA), K63‐linkage‐specific polyubiquitin D7A11 (rabbit monoclonal, Cell Signaling Technology, Danvers, MA),K43‐linkage polyubiquitin (rabbit polyclonal, Cell Signaling Technology) ClCN2 (mouse monoclonal, Sigma) or ClCN2 S787 (rabbit polyclonal antisera, Schiffhauer et al. 2013). Actin (rabbit polyclonal, Sigma) or GAPDH (mouse monoclonal Aviva Systems Biology, San Diego, CA) were added. Secondary antibodies included ECL™ Anti‐Rabbit or Anti‐Mouse IgG Horseradish Peroxidase linked antibody from donkey (GE Healthcare Life Sciences, Pittsburgh, PA).

### Immunolocalization and confocal microscopy

Transwell insert filters were fixed with 4% paraformaldehyde and acetone:methanol for 10 and 2 min, respectively. After a series of washes, the filters were blocked with 3% BSA in PBS for 30 min at room temperature.

The filters were incubated with a fluorescent‐labeled secondary antibody specific for FLAG (Alexa(R) 488 Conjugate, Cell Signaling Technology). The sections were counterstained with DAPI (4=,6‐diamidine‐2=‐phenylindole dihydrochloride) to identify nuclei. Negative controls were treated similarly but without primary antibody against FLAG (data not shown). Slides were viewed and digital photography performed with a Zeiss LSM 700 meta confocal microscope using a _40/1.3 oil immersion objective.

### Coimmunoprecipitation

Coimmunoprecipitation was performed as described previously (Schiffhauer et al. 2013). Proteins were precipitated from CFBE cell lysates by rotating sample lysates with 1.5 *μ*g FLAG or ENaC*γ* antibodies and protein A/G sepharose beads. The samples were centrifuged and eluted. Samples were electrophoresed as per normal immunoblot protocol described above.

### Lipid Raft Disruption by methyl‐beta cyclodextrin (M*β*CD)

Cells were cultured as described above and treated with 10 mmol/L M*β*CD (Sigma) on the apical surface for 30 min at 37°C. Cells were then subjected to standard biotinylation or coimmunoprecipitation protocols as described above.

### Statistical analysis

Significance was determined by using a two‐tailed Student's *t*‐test. Data are shown as mean of all replicates ± SE. All experiments shown have a minimum *n*‐value of 3.

## Results

### The C‐terminal region of ClCN2 is important for protein targeting to the apical membrane in CFBE cells

**Figure 1.**
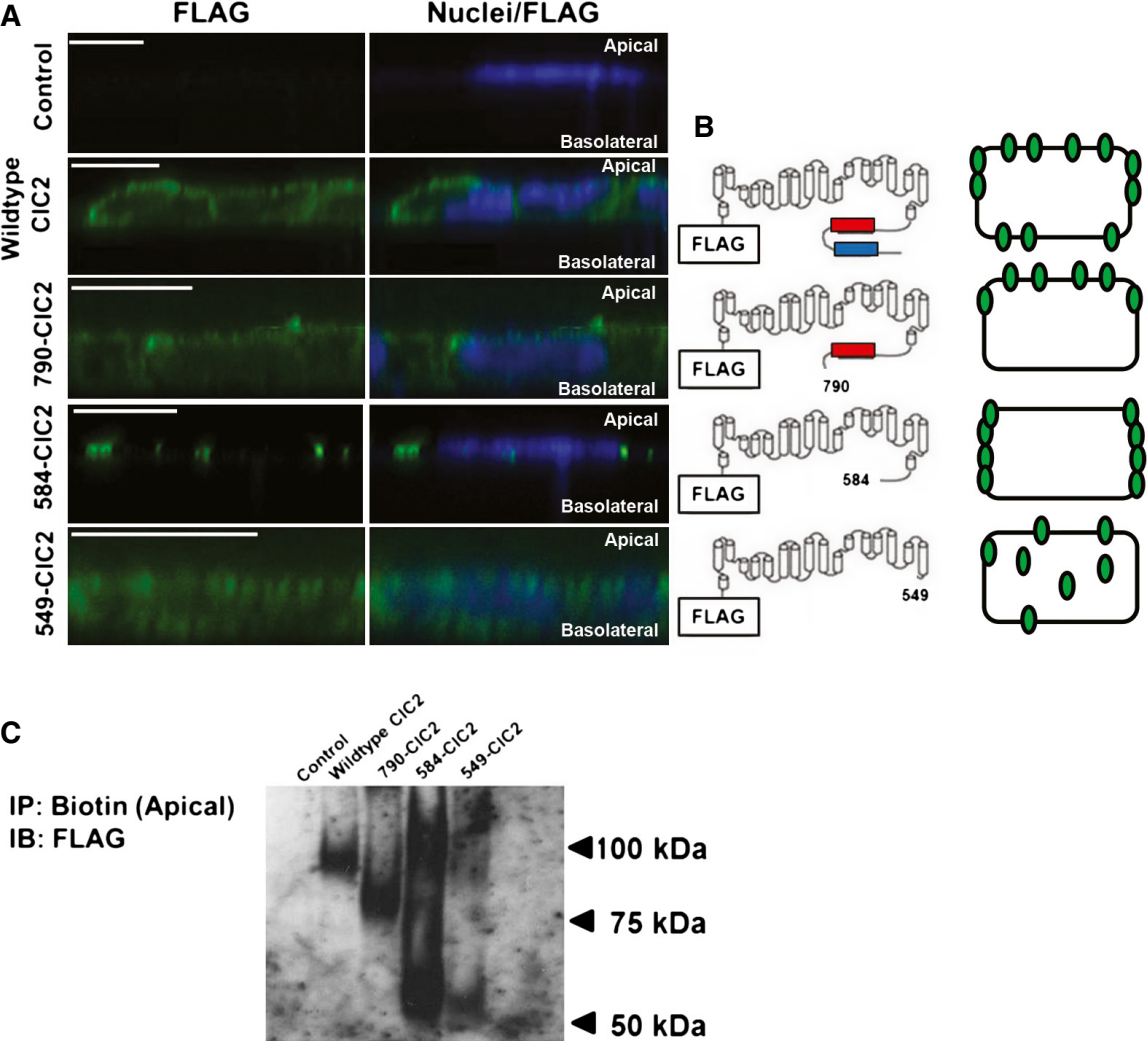
ClCN2 c‐terminus regulates protein localization, in vitro. CFBE cells transfected with FLAG‐tagged ClCN2 mutants were immunostained for FLAG (green) and nuclei (blue). Z‐stack line scans show FLAG‐ClCN2 localization for each transfection. Corresponding schematics for each mutant (right) visualize c‐terminus truncation, where red represents the ClCN2 CBS1 domain and blue represents the ClCN2 CBS2 domain (A). In (B), Schematics for each mutant visualize potential changes in ClCN2 localization. In (C) Immunoblot analysis of biotinylated proteins from the apical surface of transfected CFBE cells, where biotin is immunoprecipitated and probed for FLAG. Mutants appear at approximate corresponding weights for each truncation (98 kDa, 87 kDa, 64 kDa and 60 kDa, respectively). All bars in A are 10 *µ*m, *n* = 3.

A series of progressively truncated FLAG‐tagged ClCN2 mutants with abbreviated lengths of the c‐terminus were created by removing the CBS2 domain (790‐ClC2), CBS1 and CBS2 domains (584‐ClC2) and the entire c‐tail (549‐ClC2) (Fig. 1A, right, modified from Garcia‐Olivares et al. 2008). In order to determine any changes in ClCN2 targeting, we directly compared expression patterns of the mutants with the full‐length FLAG‐tagged ClCN2 (WT‐ClC2, Fig. 1A, right). The surface and subcellular localization of ClCN2 was visualized using transverse line scans of CFBE cells transiently transfected with each construct and immunostained for FLAG, using a FLAG‐specific secondary antibody (green) and nuclei (dapi). Visual inspection of these scans demonstrates a progressive loss of apical targeting, as demonstrated by IF staining for FLAG‐ClCN2 as larger portions of the c‐tail are removed (Fig. 1A, left). Interestingly, however, visualization of 584‐ClC2 appears to show the increased expression of this particular mutant in areas of cell–cell contact, as opposed to the apical membrane. This is summarized in a schematic, in which we speculate that loss of the CBS2 domain leads to increased localization of ClCN2 at the apical surface (Fig. 1B). Localization of ClCN2 at the apical surface was confirmed by an alternative methodology where apical plasma membranes were labeled by biotinylation, isolated and purified away from the rest of the cell and probed for FLAG (Fig. 1C) on immunoblots. In contrast to the immunofluorescent staining in Figure 1A, which does not target a particular cell surface, the biotinylation analysis of Figure 1C exclusively focuses on apical expression of ClCN2. By this analysis we confirm that the CBS1 domain is important for the targeting of ClCN2 to the apical cell surface and that when CBS2 is also present and exposed, ClCN2 expression is seen at both the apical and basolateral surfaces.

### ClCN2 regulates apical and total ENaC*γ* expression in CFBE cells

**Figure 2.**
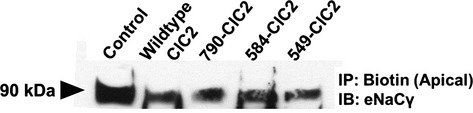
ClCN2 mediates apical localization of ENaC*γ*, in vitro. Immunoblot analysis of biotinylated proteins from the apical surface of transfected CFBE cells, where biotin is immunoprecipitated and probed for ENaC*γ* (90 kDa), *n* = 3.

The presence of ENaC at the apical surface is necessary for the absorption of Na^+^ across the cell membrane. In order to determine if changes in ClCN2 targeting can regulate the expression of ENaC, we used a biotinylation assay to determine any changes in the apical expression of ENaC*γ*, following progressive truncation of the c‐terminus of ClCN2 and subsequent changes in its membrane targeting (Fig. 1). Immunoblot analysis of biotinylated proteins from the apical surface show there is a decrease in ENaC*γ* expression at the apical cell surface when ClCN2 is increased, compared to CFBE cells transfected with a vehicle control. Interestingly, although there is a continued trend for decreased apical expression of ENaC*γ* in the presence of truncated ClCN2, there does not appear to be any significant changes in apical ENaC*γ* expression when the mutant ClCN2s are compared to WT‐ClC2 (Fig. 2). These data suggest there may be additional regions of ClCN2 beyond its potential targeting sequences that may help to regulate expression of ENaC at the cell surface.

**Figure 3.**
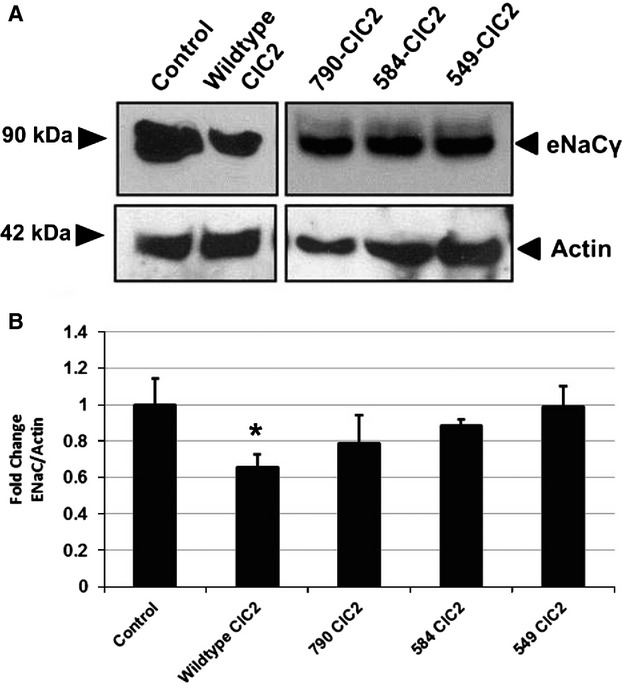
ClCN2 mediates ENaC*γ* expression, in vitro. CFBE cells were transfected with FLAG‐tagged wild‐type ClC‐2 and truncation mutants and immunoblot analysis was performed for total ENaC*γ* (90 kDa) on whole‐cell lysates, actin was used to confirm equal loading. Quantification of changes in total ENaC*γ*, normalized to actin (B) *P ≤ 0.05, *n* = 5.

Decreased expression of ENaC*γ* at the apical surface in response to increases in ClCN2 suggests a possible internalization of the sodium channel. The internalization of cell surface proteins typically results in degradation of the protein; therefore we used immunoblot analysis of whole‐cell lysates from CFBE cells transfected with wild‐type or mutant ClCN2 to determine if the decrease in apical ENaC*γ* was indicative of a loss in whole‐cell ENaC*γ* as well (Fig. 3A). Similar to changes in the apical expression of ENaC*γ*, in response to increases in wild‐type or mutant ClCN2, there is a decrease in total ENaC*γ* expression, which is significant in response to WT‐ClC2 (Fig. 3B) only.

### Ubiquitination of ENaC*γ* in response to changes in ClCN2

**Figure 4.**
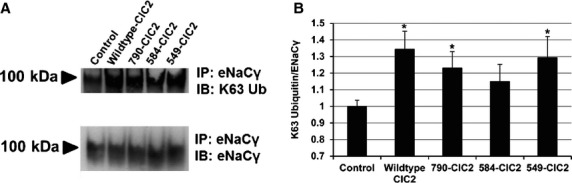
ClCN2 mediates changes in ENaC*γ* K63 ubiquitination, in vitro. ENaC*γ* was immunoprecipitated from CFBE cells transfected with wild‐type or truncated ClCN2 mutants and immunoblot analysis was performed to determine changes in ubiquitination using K63 ubiquitin (K63 Ub, 97 kDa, A). The blots were stripped and reprobed for ENaC*γ* to ensure equal immunoprecipitation of ENaC*γ* (98 kDa, A). Quantification of ENaC*γ* ubiquitination was performed normalizing K63 to immunoprecipitated ENaC*γ* (B), *P ≤ 0.05 (wild‐type ClC2) and *P ≤ 0.1 (790‐ClC2 and 549‐ClC2), *n* = 3.

Importantly, ENaC has previously been shown to be internalized from the cell surface in response to ubiquitination of the ENaC subunits, including ENaC*γ*, by Nedd4‐2 (Zhou et al. 2007; Ruffieux‐Daidie et al. 2008). With the previously mentioned decreases in both apical and whole‐cell ENaC*γ* expression, we therefore went on to measure changes in the ubiquitination of ENaC*γ* in the presence of the wild‐type or mutant ClCN2 proteins. To do so, we performed coimmunoprecipitation assays in CFBE cells transfected with vehicle control, wild‐type or mutant ClCN2 cDNA vectors. We then immunoprecipitated protein complexes with anti‐ENaC*γ* antibody, eluted the complex proteins and separated them on SDS‐PAGE gels, and then probed for ubiquitination of ENaCƔ using a K63 or K48 (data not shown) linkage‐specific antibody. The loading control was total ENaC*γ* pulled down on the beads (Fig. 4A). In response to transfection with either wild‐type or a mutant ClCN2, there was a trend for increased ubiquitination of ENaC*γ* by a K63 linkage for all four ClCN2 mutants, which was significant for WT, 790, and 549 ClCN2 mutants when compared to CFBE cells transfected with a vehicle control, suggesting decreases in ENaC expression, both overall and at the apical cell surface may be due in part to degradation of the protein, as initiated by ubiquitination of K63‐linked ubiquitin (Fig. 4B). In contrast, ubiquitination by a K48 linkage showed no apparent association with changes in ClCN2 expression (data not shown). Therefore, any therapeutic upregulation of ClCN2 in cystic fibrosis should deplete apical ENaC*γ*, thereby tempering sodium regulation, a desirable outcome in CF airways disease. It is currently unknown whether activation of chloride transport through ClCN2 plays any additional role in this downregulation of sodium reabsorption.

### ClCN2 physically interacts with ENaC*γ*

**Figure 5.**
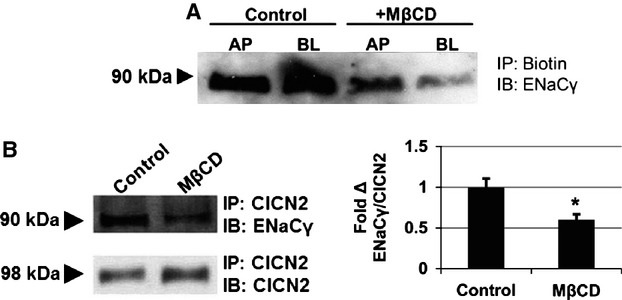
Physical interaction between ClCN2 and ENaC*γ* dependent on protein proximity at the apical surface. Immunoblot analysis of biotinylated proteins from the apical and basolateral surface of CFBE cells from control or M*β*CD‐treated conditions, probed for ENaC*γ* (A). In B, Endogenous ClCN2 is immunoprecipitated from CFBE cells treated with M*β*CD and immunostained for ENaC*γ*, stripped and reprobed for ClCN2 as a loading control (B, left). Quantification shows the fold change of coimmunoprecipitated ENaC*γ* between control and M*β*CD conditions (B, right). *P ≤ 0.05, *n* = 3.

Given the interregulation between the cystic fibrosis transmembrane regulator CFTR and the epithelial sodium channel ENaC, we investigated the possibility that ClCN2 directly associates with a subunit of ENaC. The ability of endogenous ClCN2 to physically interact with ENaC*γ* was first confirmed using a coimmunoprecipitation assay in polarized CFBE cells under control conditions. The importance of apical expression of ENaC*γ* for the interaction between the two proteins was then assessed following disruption of lipid rafts using M*β*CD. As expected, ENaC*γ* was present at the apical surface (Fig. 5A), which coincided with a strong interaction between it and ClCN2 (Fig. 5B). Biotinylation assays demonstrated a loss of ENaC*γ* at the apical surface, following lipid raft dissolution by M*β*CD (Fig. 5A), Disruption of the lipid rafts led to a significant decrease in the interaction between ClCN2 and ENaC*γ* (Fig. 5B), suggesting the presence of ENaC at the apical surface is an important factor for the interaction between the two proteins. Interestingly, ENaC*γ* was also shown to be at the basolateral surface as well, although there is little evidence to support ENaC*γ* activity at the basolateral surface at this time, it is possible its presence may play some role in regulation of ion movement.

**Figure 6.**
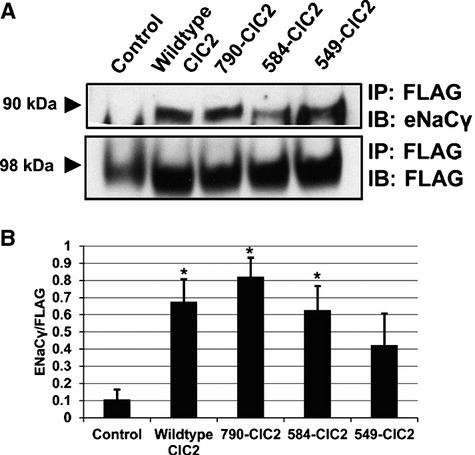
ClCN2 interacts with ENaC*γ*, in vitro. FLAG‐tagged ClCN2 mutants are immunoprecipitated from transfected CFBE cells and immunoblot analysis was performed for ENaC*γ* (90 kDa). The blots were stripped and reprobed for FLAG to ensure equal loading (98 kDa, A). Quantification of ENaC*γ* coimmunoprecipitation, normalized to FLAG (B). *P ≤ 0.01 (Wild‐type ClC2, 790‐ClC2) *P ≤ 0.05 (584‐ClC2), *n* = 4.

To confirm the importance of apical colocalization of ENaC*γ* and ClCN2 in the physical interaction between the two, we transfected polarized CFBE cells with the ClCN2 mutants and again used coimmunoprecipitation assays to determine if apical targeting of ClCN2 by the c‐tail was important in the ability of ClCN2 and ENaC*γ* to physically interact (Fig. 6A). By immunoprecipitating with an antibody specific to FLAG, we can immunoprecipitate each mutant and by probing for ENaC*γ*, we are then able to show any changes in the interaction between ClCN2 and ENaC*γ* as being the result of changes in the ClCN2 c‐terminus, such as its targeting to the apical cell surface. We show there is an apparent decrease in the interaction between ClCN2 and ENaC*γ* with the loss of the CBS‐1 domain from ClCN2, which is significantly less when WT ClCN2 was compared to the 790 and 584 ClCN2 mutants and quantified using each FLAG pulldown as a loading control for its respective mutant paradigm **(**Fig. 6B**)**.

## Discussion

During cystic fibrosis, the loss of functional CFTR leads to a disruption in the regulation of ion channels at the cell membrane, namely, the negative regulation of sodium absorption through the apically located sodium channel, ENaC (Riordan 1999; Gentzsch et al. 2010). Recent efforts to identify alternative chloride channels for potential therapeutic targets highlight the chloride channel ClCN2 as having similar expression patterns and functions as CFTR (Schiffhauer et al. 2013). Although the targeting of ClCN2 for the restoration of Cl^−^ secretion is under investigation (Schiffhauer et al. 2013), we chose to look at the potential role of apical targeting of ClCN2 in the direct negative regulation of ENaC at the apical surface of lung epithelium. By doing so, we hope to elucidate components of the regulatory mechanism, so as to better provide more specific options for therapeutic targets in the future.

During CF, the increase in sodium absorption occurs namely through increased activity of the apically expressed protein, ENaC (Riordan 1999; Gentzsch et al. 2010). It is therefore likely that the concurrent expression of ClCN2 at the apical surface would be important for its ability to regulate ENaC by a physical interaction. The exact localization of ClCN2 in lung epithelium is not agreed upon, but it has been suggested that the expression of ClCN2 is dependent on possible targeting sequences located within the c‐terminus of the protein, which also contains two CBS domains (Pena‐Munzenmayer et al. 2005). We chose to exploit this potential targeting mechanism and created FLAG‐tagged ClCN2 mutants with truncated portions of the c‐terminus (Fig. 1A, right). These truncations removed either one or both of the CBS domains or the entire c‐terminus. A FLAG‐tagged wild‐type ClCN2 was also created for comparison. Complete truncation (549 ClCN2), as truncation of both CBS domains of the c‐terminus resulted in decreased ClCN2 targeting to the apical surface. However, WT‐ClC2 and to a lesser extent the 790 and 584 mutants were strongly expressed in the apical surface of CFBE cells (Fig. 1), confirming a targeted increase in ClCN2 can result in the colocalization of ClCN2 and ENaC in CF cells, providing increased opportunity for the direct regulation of ENaC at the apical surface. There does appear to be the presence of multiple bands for the ClCN2 mutants at the apical surface. While we are unable to directly identify these, it is interesting to speculate that various levels of posttranslational modification may be occurring on the separate mutants. Understanding what changes in posttranslational modifications, as well as their downstream effects on ClCN2 may have profound impacts on the future research of CF therapeutics. Interestingly, unlike loss of the entire c‐terminus, loss of both CBS domains resulted in accumulation of ClCN2 at areas cell junctions which suggests this small portion of ClCN2 may present a separate function in CF unrelated to ENaC regulation. These data are especially interesting when considering publications which show an important role for ClCN2 in the regulation of tight junction formation (Moeser et al. 2007). Therefore, although it is not within the scope of this current manuscript, it is intriguing to consider what role potential changes in tight junction regulation by ClCN2 may play in CF therapeutics. Our results further suggest that ClCN2 may be present at both the apical and basolateral surfaces in the lung epithelium. It is also possible that the CFBE cell line is not a perfect representation of an in vivo tubular epithelium. Although it is not the current focus of this work, the potential role of basolateral ClCN2 in the treatment of CF is unknown and is the subject of continuing interest in our laboratory.

In order to further define the potential mechanism for negative regulation of ENaC by ClCN2, we also looked at changes in the expression of ENaC*γ*. Interestingly, a decrease in ENaC*γ* at the apical surface is seen when WT‐ClC2 is increased (Fig. 2) suggesting the increased presence of ClCN2, especially at the apical surface, may result in the internalization of ENaC from the cell surface. Protein internalization is typically initiated by an ubiquitination process, where ubiquitin proteins are attached to the protein of interest, thereby marking it for internalization and ultimately protein degradation (Hicke 1997, 2001; Hershko and Ciechanover 1998). Indeed, ENaC*γ* has been shown to be ubiquinated via a Nedd4‐2‐mediated mechanism, a process which ultimately leads to the degradation of ENaC protein (Zhou et al. 2007; Ruffieux‐Daidie et al. 2008). Under our experimental conditions, we also show that not only is there a decrease in apical expression of ENaC*γ* when apical ClCN2 is increased, but that this decrease occurs concurrent with a decrease in total expression of ENaC*γ* as well (Fig. 3). These data suggest that the internalization of ENaC from the apical surface may be the result of an ubiquitination mechanism that leads to the degradation of ENaC protein when increased ClCN2 is present. In support of this theory, we show that compared to control conditions, increases in ClCN2 do appear to increase ENaC*γ* ubiquitination (Fig. 4). Interestingly, although there is a trend for increased ubiquitination of ENaC*γ* in response to all four ClCN2 mutants, 584‐ClC2 ubiquitination remains insignificant compared to control conditions, despite a significant increase in binding to ENaC*γ*. The cause for this discrepancy is currently unknown, although it is possible that the 584‐ClC2, is more prominently expressed in areas of cell–cell interactions, thereby changing the nature of its interaction with ENaC at the cell surface. The differential roles for ClCN2 at the apical surface versus areas of cell–cell interaction could provide additional therapeutic targeting in the treatment of CF and is the subject of ongoing experiments in the laboratory. In sum, these data would suggest that ClCN2 may regulate expression of ENaC at the apical surface by promoting internalization and ultimately, degradation of the protein, possibly by ubiquitination.

This promotion may in turn be regulated by the ability of ClCN2 to bind to ENaC at the cell surface. Possible mechanisms of action for this could include exposure of ubiquitination sites on ENaC ubiquitination once bound by ClCN2 or alternatively, as seen with CFTR, binding of ClCN2 to ENaC may prevent the proteolytic activation of ENaC (Berdiev et al. 2007; Gentzsch et al. 2010), thus allowing for the balance of degradation versus activation to shift in the favor of ENaC internalization (Ruffieux‐Daidie et al. 2008). It is important to note that the ENaC channel in lung epithelium is comprised of three subunits, *α*,* β,* and *γ*, although preliminary data from our laboratory, which is not shown here, does suggest changes in response to ClCN2 are occurring in all three ENaC subunits. Furthermore, it is generally accepted that the channel must have three functioning subunits in order to be active. With this in mind, we have chosen to focus on ENaC*γ* due to consistency in antibody performance, however, identifying equally as reliable reagents to study the remaining two ENaC subunits is an ongoing focus in our laboratory, which may in turn provide additional means for therapeutic exploitation.

One of the most common forms of protein regulation by another protein is a direct physical interaction between the two. Although a mechanism of action for the regulation of ENaC by CFTR has yet to be defined, it has been suggested that the two proteins may physically interact as one means of channel regulation (Berdiev et al. 2007; Gentzsch et al. 2010). We therefore wanted to determine if ClCN2 may regulate ENaC by a physical interaction, which could result in inhibition of ENaC activity as well as exposure of ubiquitination sites on ENaC, especially ENaC*γ*. Our data support a physical interaction, as we are the first to show that in CFBE cells, there is a physical interaction between endogenous ClCN2 and ENaC, specifically ENaC*γ* (Fig. 5). In nontransfected cells, disruption of lipid rafts by M*β*CD led to a marked decrease in ENaC*γ* at the apical surface, which resulted in a decrease in the physical interaction between ClCN2 and ENaC*γ*, (Fig. 2). These data appear to support the importance of the two proteins being coexpressed at the apical surface in order for a physical interaction to occur. Interestingly, these data also suggest the presence of ENaC*γ* at the basolateral surface, which is also diminished in response to M*β*CD (Fig. 5). While it is unlikely that the regulation of ENaC by ClCN2 is occurring at the basolateral membrane, there are some data to suggest that ENaC may be trafficked to the apical surface by way of the basolateral membrane (Butterworth 2010). As the trafficking of ENaC is thought to be regulated by lipid rafts (Hill et al. 2002, 2007; Butterworth 2010), it would therefore follow to see a decrease in ENaC*γ* expression at the basolateral surface following the disruption of lipid rafts by M*β*CD as seen in our data. It is also important to note that CFBE have low endogenous ENaC activity. As such, it is interesting to speculate that this absence of appreciable ENaC current may be due in part to a high level of ENaC expression at the basolateral surface. These considerations articulate the intricacies of ion channel regulation and highlight the importance of learning how ClCN2 targeting may emphasize interactions with apical versus basolateral ENaC in CFBE cells.

The importance of proximity between ENaC*γ* and ClCN2 at the apical surface was reinforced by showing this novel interaction can also be manipulated based on levels of ClCN2 expression at the apical surface, which we show using ClCN2 c‐terminus truncation mutants (Fig. 6). As such, our data seem to suggest that the interaction between ClCN2 and ENaC*γ* is largely dependent on the proximity of the two proteins in the apical membrane of the lung epithelium. That is to say, that when loss of ClCN2 targeting to the apical surface occurs, as seen with the 549‐ClC2 and to some extent the 584‐ClC2 mutant, there is a decrease in the observed interaction between ClCN2 and ENaC*γ*. Alternatively, when there is an increase in ClCN2 at the apical surface, as seen with the WT‐ClC2 and 790‐ClC2 transfected cells, the interaction between ClCN2 and ENaC*γ* increases dramatically. These data therefore indicate that the c‐terminus of ClCN2, especially the CBS‐1 domain, may be important for the regulation of ENaC by ClCN2. Our data would suggest that the targeting of ClCN2 to the apical surface is dependent on the presence of the CBS1 domain, whereas portions of the c‐terminus above the CBS1 domain may be important for the regulation of cell junctions by ClCN2. Further work is required to elucidate the exact importance of the c‐terminus in the physical interaction between ClCN2 and ENaC at the apical surface of lung epithelium as a result of ClCN2 targeting, as well as the potential pleiotropic role for ClCN2 in the regulation of cell junctions. It is also important to note that although we do observe a physical interaction between ClCN2 and ENaC*γ* which changes in response to ClCN2 truncation, portions of the c‐terminus are also suggested to play an important role in the gating of ClCN2 (Ramjeesingh et al. 2006). Changes in the gating of ClCN2 may lead to a shift in the ionic balance across the membrane, thus regulating the driving force for Na^+^ absorption by ENaC in an indirect manner, similar to what has been reported for CFTR (Briel et al. 1998).

Our data suggest that in response to changes in ClCN2 expression, there is a marked shift in ENaC*γ* expression at the apical surface of lung epithelium, in vitro. Specifically, we identify for the first time a novel physical interaction between ClCN2 and ENaC*γ*, as well as a possible regulation mechanism involving the internalization and degradation of ENaC*γ* by ubiquitination. Although the data discussed are performed exclusively in vitro, they provide important insight into understanding how ENaC and ClCN2 may interact in vivo, as well as how these interactions can be further studied in future, more physiologically relevant studies currently underway in the laboratory. In sum, these data would seem to support the ability of ClCN2 to negatively regulate Na^+^ absorption by ENaC in a direct manner during CF and provide valuable mechanistic targets for the further therapeutic treatment of CF.

## Conflicts of Interest

No conflicts of interest, financial or otherwise, are declared by the author(s).
